# Xylitol and erythritol inhibit real-time biofilm formation of *Streptococcus mutans*

**DOI:** 10.1186/s12866-020-01867-8

**Published:** 2020-06-29

**Authors:** Vuokko Loimaranta, Danuta Mazurel, Dongmei Deng, Eva Söderling

**Affiliations:** 1 Institute of Dentistry, University of Turku, Lemminkäisenkatu 2, 20520 Turku, Finland; 2 Department of Preventive Dentistry, Academic Centre for Dentistry Amsterdam (ACTA), University of Amsterdam and VU University Amsterdam, Amsterdam, Netherlands

**Keywords:** Xylitol, Erythritol, *Streptococcus mutans*, Biofilm, Polysaccharides

## Abstract

**Background:**

Regular consumption of xylitol decreases the number of cariogenic streptococci in dental plaque. In vitro biofilm models to study the mechanism of xylitol action have been set-up, but the obtained results are contradictory. Biofilm growth is a dynamic process with time-specific characteristics that may remain undetected in conventional end-point biofilm tests. In this study we used an impedance spectroscopy instrument, xCELLigence Real Time Cell Analyzer (RTCA), that allows label-free, non-invasive real-time monitoring of biofilm formation, to explore effects of xylitol on biofilm formation by *Streptococcus mutans*. Based on the obtained information of biofilm dynamics, we assessed the number of viable bacteria, the polysaccharide content, and the expression levels of selected genes involved in glucan-mediated biofilm formation in different biofilm stages. Xylitol inhibition was compared with that of erythritol; another polyol suggested to have a positive impact on oral health.

**Results:**

Our results showed that real-time monitoring provided new information of polyol-induced changes in *S. mutans* biofilm formation dynamics. The inhibitory effect of polyols was more pronounced in the early stages of biofilm formation but affected also the measured total amount of formed biofilm. Effects seen in the real-time biofilm assay were only partially explained by changes in CFU values and polysaccharide amounts in the biofilms. Both xylitol and erythritol inhibited real-time biofilm formation by all the nine tested *S. mutans* strains. Sensitivity of the strains to inhibition varied: some were more sensitive to xylitol and some to erythritol. Xylitol also modified the expression levels of *gbpB*, *gtfB, gtfC* and *gtfD* genes that are important in polysaccharide-mediated adherence of *S. mutans*.

**Conclusion:**

The erythritol- and xylitol- induced inhibition of biofilm formation was only partly explained by decrease in the number of viable *S. mutans* cells or the amount of polysaccharides in the biofilm matrix, suggesting that in addition to reduced proliferation also the matrix composition and thereby the surface attachment quality of biofilm matrix may be altered by the polyols.

## Background

Dental caries results from microbiome dysbiosis involving multiple cariogenic bacterial species, including *Streptococcus mutans* and *S. sobrinus* (mutans streptococci, MS [1, 2];)*.* The relevancy of MS in the etiology of dental caries has been questioned [3]. MS have, however, key pathogenic properties as prominent extracellular polysaccharide (EPS) matrix producers, and as acidogenic and aciduric organisms [1–4]. Extracellular polysaccharides (EPS) in dental plaque could predict caries development in children [5]. In addition, high levels of MS in the dentition appears also to be one of the strongest risk indicators associated with early childhood caries [6, 7].

Xylitol and erythritol are five- and four-carbon polyol sweeteners, respectively, that appear to have specific, beneficial effects on oral health [8–11]. Earliest studies of xylitol inhibition of MS are reported already at 1975 [12], but erythritol has only recently become a subject of wider interest [11]. Habitual consumption of xylitol is suggested to reduce caries occurrence, plaque amount and levels of MS [8–10]. Habitual, long-term xylitol consumers are also reported to have low levels of dental plaque compared to non-consumers of xylitol [13]. It has been suggested that “xylitol-plaque” is less adhesive due to a decrease in counts of plaque mutans streptococci and the amount of insoluble polysaccharides in plaque [9, 14]. Erythritol has been suggested to outshine xylitol with regard to oral health benefits [11]. There are, however, only a few clinical studies evaluating the effects of erythritol on oral health-related variables. Erythritol appears to decrease plaque amount, but the effects on MS levels are controversial [15–17].

In spite of the abundant research published, the mechanism of the plaque-decreasing effect of xylitol is not fully understood. The decrease in plaque MS counts is often attributed to growth inhibition, but also to an extracellular polysaccharide-mediated decrease in plaque adhesiveness [9]. To explore the mechanisms of xylitol inhibition of biofilms, several in vitro studies are conducted, but the results are contradictory, both inhibition [18–21] or no effect on MS in biofilm has been reported [18, 22–24]. Two studies on effects of erythritol on *S. mutans* biofilm formation on polystyrene microtiter plates were identified, both showing inhibition on total amount of biofilm formed by *S. mutans* [20, 25].

Comparison of the in vitro studies is difficult because different bacterial strains and conditions are used and different parameters, e.g. number of bacteria, total biomass of biofilm, amount of EPS, measured. Moreover, the used biofilm set-ups are mostly end-point measurements and obtained information is thus limited to the selected biofilm stage. Biofilm growth, however, is a dynamic time-dependent process with time-specific characteristics. It has been, for example, suggested that xylitol reduces the number of viable *S. mutans* bacteria in the early stages but not in mature biofilm [19].

In this study we aimed to follow the effects of xylitol on the dynamics of biofilm formation by different strains of *S. mutans* using an impedance spectroscopy instrument, xCELLigence Real Time Cell Analyzer (RTCA) from Acea Biosciences, that allows label-free, non-invasive real-time monitoring of biofilm formation [26, 27]. Based on the obtained information of biofilm dynamics, we assessed the number of viable bacteria, the polysaccharide content, and the expression levels of genes involved in glucan mediated biofilm formation in different biofilm stages of selected strains. Comparative studies of the effects of xylitol and erythritol on *S. mutans* biofilms are almost non-existent [20, 28] thus, we also explored the effects of erythritol on *S. mutans* biofilm dynamics.

## Results

### Biofilm formation

**Fig. 1:**
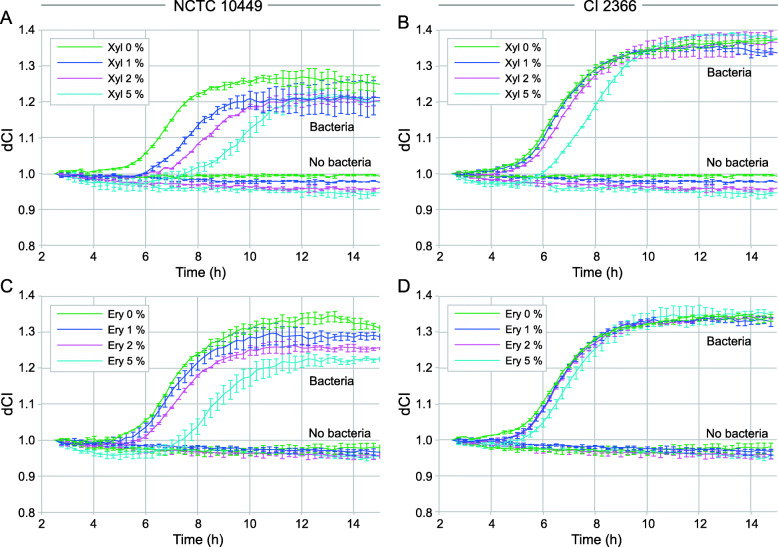
Real-time biofilm formation of (**a**) *S. mutans* NCTC 10449 and (**b**) clinical isolate 2366 in BHI-sucrose medium or medium supplemented with 1, 2% or 5% xylitol and (**c**) S. mutans NCTC 10449 and (**d**) clinical isolate 2366 in BHI-sucrose medium or medium supplemented with 1, 2% or 5% erythritol. Bacteria were let to adhere on E-plate surface for 2 h, after which xylitol was added in the medium. dCI = cell index value change after addition of xylitol, see text for details. Mean ± SD, *n* = 4

In preliminary experiments different concentrations of xylitol and erythritol (1, 2, 5%) were tested. Dose-dependent reduction in the CI was observed in xylitol-supplemented BHI-sucrose medium (Fig. 1a and b). The biofilm formation of the reference strain NCTC 10449 was inhibited even with 1% xylitol while the inhibition of biofilm formation of the strain 2366 required 5% of xylitol. Similar results were seen with erythritol (Fig. 1c and d). The biofilm formation appeared to reach maximum CI-values between 10 and 12 h. For the strain 2366 the tested polyols reduced the rate of biofilm formation, but did not affect the total biomass of formed biofilm, as measured by RTCA (Fig. 1b and d). For the strain NCTC 10449, instead, the total biomass of formed biofilm was significantly reduced by the presence of both xylitol and erythritol (Fig. 1a and c). Based on these results, 5% xylitol and erythritol were selected for tests with further strains.

**Fig. 2:**
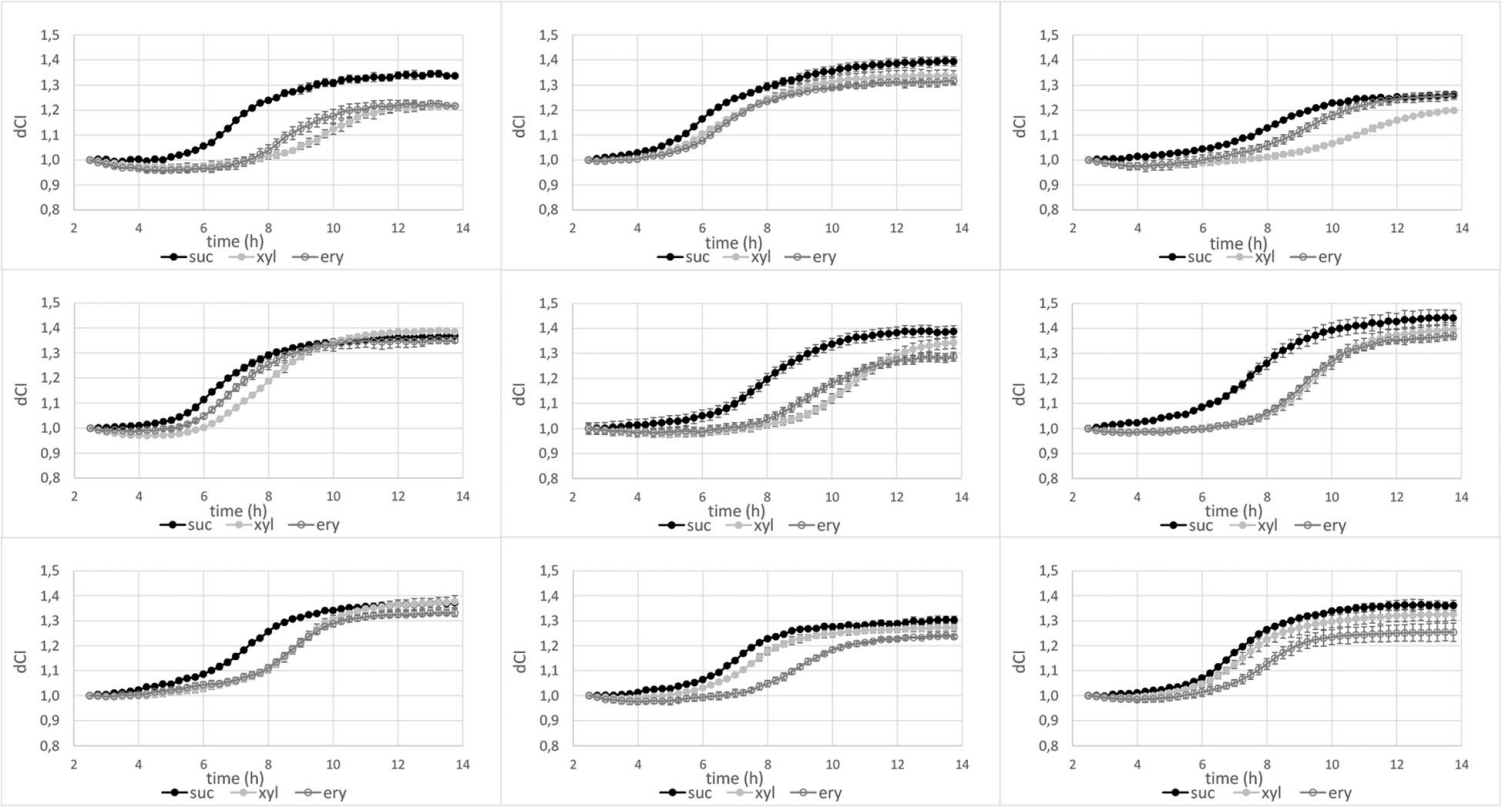
Real-time biofilm formation of different *S. mutans* strains in the BHI-sucrose medium (suc) or medium supplemented with 5% xylitol (xyl) or 5% erythritol (ery). **a***S. mutans* NCTC10449 **b***S. mutans* NG8 **c***S. mutans* Ingbritt **d***S. mutans* 2366 **e***S. mutans* 195-s2 **f***S. mutans* 199-s6 **g***S. mutans* 124-s7 **h***S. mutans* 113-s12 **i***S. mutans* 117-s3. Bacteria were let to adhere on E-plate surface for 2 h, after which xylitol was added in the medium. dCI = cell index value change after addition of xylitol, see text for details. Mean ± SD, *n* = 2–4

The effect of the polyols on the biofilm formation of nine different *S. mutans* strains were tested, three reference strains *S. mutans* NCTC 10449, *S. mutans* NG8 and *S. mutans* Ingbritt as well as six clinical isolates (Fig. 2). All tested strains were inhibited by the polyols but the sensitivity varied significantly between strains, some were more susceptible for xylitol, some for erythritol while for some the effects of the polyols were almost indistinguishable. For all strains and for both polyols the inhibition was most evident at early stage of biofilm formation. (Fig. 2).

To get information of the parameters responsible of measured differences in biofilm formation, *S. mutans* NCTC 10449 and CI2366 biofilms were analysed in more detail. Viable bacteria, amount of extracellular carbohydrates in the biofilm and the expression of *gbpB-* and *gtf* -genes were measured at different time points.

### Colony forming units

**Fig. 3:**
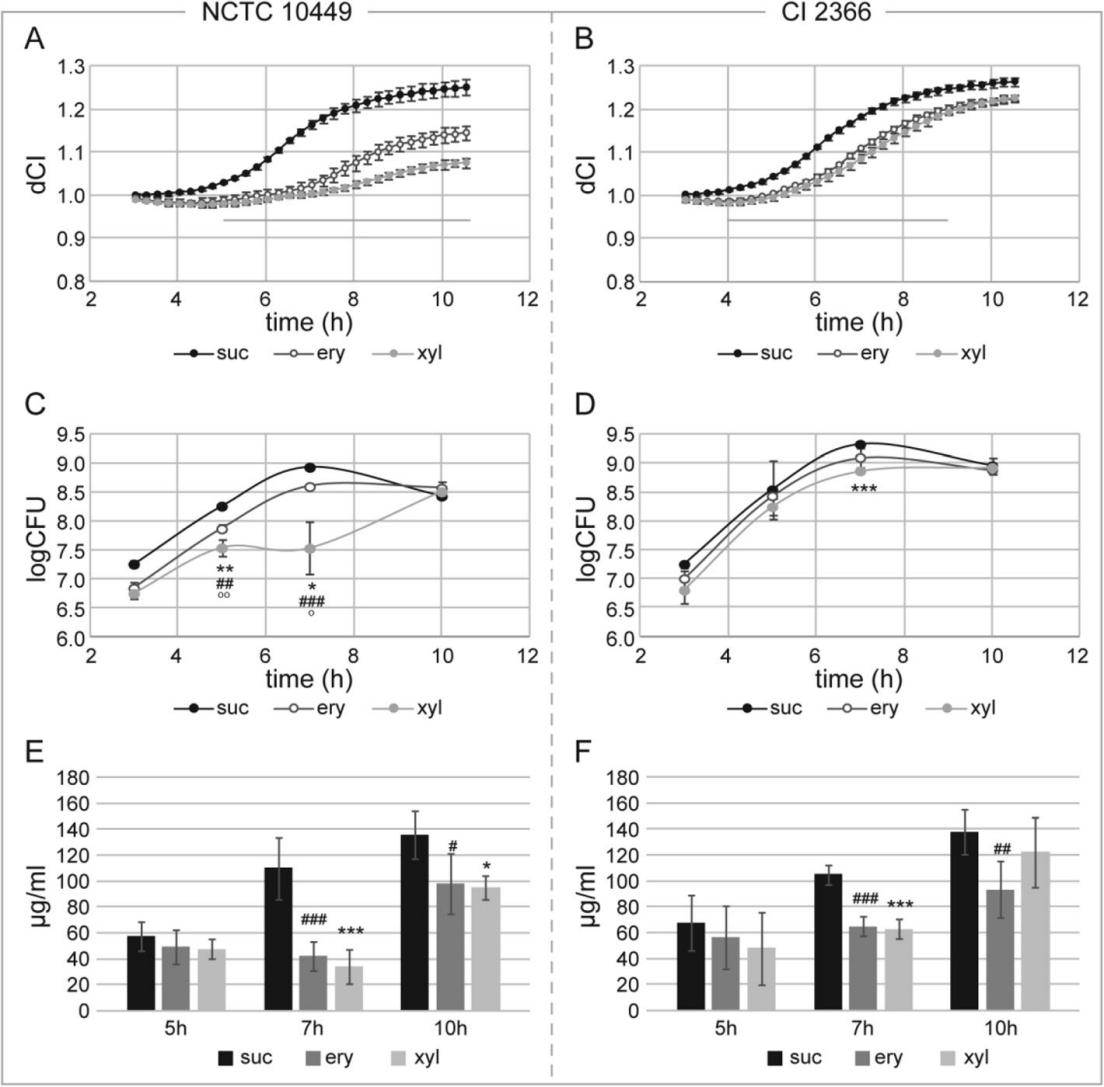
Formation rate and composition analysis of biofilms made by *S. mutans* NCTC 10449 (**a, c, e**) and clinical isolate 2366 (**b, d, f**) in BHI-sucrose medium (suc), and BHI-sucrose medium supplemented with eryhtritol (ery) or xylitol (xyl). **a** and **b**: Biofilm formation as measured in xCELLigence, the erythritol and xylitol curves significantly differed from sucrose in all time points, the line indicates the time period where there is a significant difference (*p* < 0.05) between erythritol and xylitol, **c** and **d**: Biofilm samples were collected at indicated time points and number of living bacteria were determined by plate counting, **d** and **e**: Amount of polysaccharides in the biofilm at different time points as measured by Anthrone method from collected biofilm samples. * significant difference (*p* < 0.05) between sucrose and xylitol, ^#^significant difference (*p* < 0.05) between sucrose and erythritol, ^o^significant difference (*p* < 0.05) between erythritol and xylitol, **/^##^/^oo^*p* < 0.01, ***/^###^/^ooo^*p* < 0.001

The number of viable bacteria in the biofilm formed under polyol exposure was reduced in the early phases of the biofilm formation by both tested strains (Fig. 3c and d). The presence of xylitol inhibited the growth of *S. mutans* NCTC 10449 more than erythritol, especially at seven-hour time point (*p* = 0.016, Fig. 3c). However, no difference was noted in the biofilm viability at ten-hour time point even though the amount of formed biofilm was significantly reduced by erythritol and xylitol.

### Polysaccharide formation

To compare the extracellular polysaccharide matrix of the biofilms, we next measured the total amount of carbohydrates at different time points. The carbohydrate amount increased during time, as expected, and correlated well with the measured biofilm formation/CI values in E-plate when bacteria were grown in BHI-sucrose (Fig. 3a-b, and e-f).

At the seven-hour time point, the presence of polyols reduced the amount of measurable carbohydrates in biofilms formed by both tested *S. mutans* strains, when compared to biofilm formed in control BHI-sucrose medium without polyols. In the ten-hour samples, especially biofilms formed by *S. mutans* NCTC 10449 in xylitol or erythritol supplemented medium still contained significantly less carbohydrates than those formed in BHI-sucrose medium. This is in accordance to measured CI values of the biofilms, where the effect of polyols on the biofilm formation by strain NCTC 10449 was more pronounced than on biofilm formation by strain 2366 (Fig. 3a-b and e-f). No difference could be seen between erythritol and xylitol biofilms in polysaccharide formation.

### Gene expression

The expression levels of *gbpB,* encoding glucan binding protein B, an important mediator in sucrose-dependent biofilm formation [29], and three glucosyltransferase genes, *gtfB, gtfC* and *gtfD,* coding the enzymes responsible for extracellular polysaccharide synthesis, were evaluated. The strain 2366 and biofilms formed in control and xylitol supplemented medium were selected for this assay, because previous studies have shown that xylitol inhibits the growth and polysaccharide-mediated adhesion of strain 2366 more efficiently than that of the reference strain [18, 28, 30].

The overall yield of RNA extracted from xylitol biofilms at both the seven- and ten-hour time points were lower than that obtained from control biofilms. The RNA yields for the xylitol-treated biofilms were 24.5 ± 12.5 ng (7 h) and 752.2 ± 339.5 ng (10 h) and for the control biofilms 259.6 ± 212.3 ng (7 h) and 1298.9 ± 304.1 ng (10 h). This could partly be explained by lower number of bacteria present in the xylitol biofilm at the seven-hour time point, but at the ten-hour time point equal numbers of viable bacteria were present in both biofilms.

**Fig. 4:**
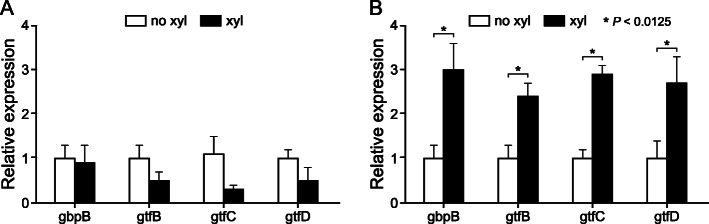
Expression of genes involved in glucose mediated adhesion and extra cellular polysaccharide formation in *S. mutans* 2366 biofilms at two different time points. **a**: 7 h, **b**: 10 h. Expression is depicted as fold change in gene expression in xylitol-biofilm in relation to biofilm formed in unsupplemented BHI-sucrose medium. * *p* < 0.0125

No effect of xylitol supplementation was seen on the expression level of *gbpB* nor the three glucosyltransferase genes, *gtfB*, *gtfC* and *gtfD* in the seven-hour biofilms but the expression of all of them was significantly upregulated in the ten-hour xylitol biofilms (Fig. 4).

## Discussion

Our results showed that both xylitol and erythritol inhibited real-time biofilm formation of the used *S. mutans* strains in the presence of 1% sucrose, but the sensitivity of the strains to polyols differed. The polyol-induced inhibition of the real-time biofilm formation was only partly explained by a decrease in the number of viable *S. mutans* cells or the amount of polysaccharides in the biofilms.

When polysaccharide production is one target of the study, addition of sucrose to the medium is essential. In the real-time biofilm monitoring method we used, *S. mutans* biofilm formation is significantly increased by sucrose in the medium, 1% being superior to 0.2% [26]. Thus, we chose to include 1% sucrose in the medium. In some cases sucrose added to the medium is reported to abolish polyol inhibition observed without added sucrose [18], but by the assay used in the present study polyol-induced inhibition in the biofilm formation could be seen for all tested strains even in the presence of sucrose.

In the literature the xylitol-induced effects on biofilm formation have shown big variation e.g. [18, 19, 21, 24, 28]. This may reflect for different experimental conditions and different bacterial strains used. Different strains have different sensitivity to xylitol and erythritol as evidenced in the present study, and also in earlier studies [18, 19, 28]. Some strains were more sensitive to xylitol and some more sensitive to erythritol, and there were also strains where the effect of polyols was almost identical. For all strains, the inhibitory effect of the polyols on the *S. mutans* biofilm growth was more pronounced in the early stages of biofilm formation and less significant at later stages. The long incubation time in the biofilm experiment may explain why, for example, Giertsen et al. [24] found no effects for repeated exposures with 7.5% xylitol in a 64.5-h-old mixed-six-species biofilm.

We could see a reduction in the number of viable bacteria in the early time point biofilms formed under both xylitol and erythritol exposure, but not in any biofilms at the ten-hour time point. Also in our earlier end-point biofilm experiment 5% xylitol inhibited *S. mutans* counts in biofilm only when the biofilm was young, 8 h old, but not at 24 h [19]. Both erythritol and xylitol inhibit the growth of planktonic *S. mutans* cells [16, 28, 30]. Interestingly, xylitol reduced the number of *S. mutans* NCTC 10449 bacteria in the biofilm more efficiently than erythritol, even though both polyols inhibit the planktonic growth of the strain equally well [16, 28]. The mechanism of erythritol inhibition is not known, but xylitol inhibits the growth of *S. mutans* via a futile xylitol-5-phosphate cycle. The inhibition is related to the PEP:PTS activity, resulting in ‘starvation’ of the cells [31]. At the ten-hour time point we noted markedly lower amounts of RNA isolated from bacteria growing in biofilms exposed to xylitol compared to RNA from similar numbers of live bacteria from control biofilms, which may reflect the xylitol-induced ‘starvation’ of the bacteria in the xylitol biofilm. Our results thus suggest that growth inhibition may contribute to biofilm inhibition at the early stages, and even though the number of viable bacteria is not reduced in later stages, their protein synthesis may be impaired.

In a clinical study, habitual exposure of plaque to xylitol decreased insoluble polysaccharides in the plaque [14]. In our in vitro model both polyols affected equally to the dynamics of the polysaccharide accumulation in the biofilm matrix of the two analysed strains. In all biofilms the biofilm amount increased up to 10–12 h. The fastest accumulation occurred between five and seven hours in the absence of the polyols while in polyol biofilms the fastest accumulation typically started 1–3 h later. Polysaccharide-mediated adhesion is an important step for the subsequent formation of mature biofilm. This process involves bacterial surface proteins, e.g. glucan binding proteins that attach to polysaccharides in the biofilm matrix, synthesized mainly by the three glucosyltransferases, GtfB, GtfC and GtfD. We therefore measured the expression levels of *gbpB* and *gtf* -genes in the strain 2366 in the presence and in the absence of xylitol. In the seven-hour samples, no difference was seen between control and xylitol biofilms in relative expression levels of *gbpB*- or *gtf-*genes. The reduced amount of measured polysaccharides in the seven-hour biofilm may reflect lower numbers of viable bacteria in the biofilm, but it is also possible that xylitol- induced starvation reduced the total protein synthesis of bacteria, not seen in qPCR which measures only the relative expression of genes. The increased *gtf-* gene expression levels seen in the ten-hour xylitol biofilms is reflected also as higher polysaccharide accumulation in xylitol biofilms compared to control biofilms during later stages of biofilm. To our knowledge, the effect of xylitol on *gbpB*- or *gtf -*gene expression in the presence of sucrose has not been studied before. Decker et al. studied *S. mutans* biofilms exposed to 1% xylitol in medium containing glucose and found upregulation of *gtfB, gtfC* and *gtfD* in 24-h biofilm, suggesting that the metabolic imbalance caused by xylitol was counteracted at the gene expression level [22]. This is in line with our results showing that effects of xylitol on *gtf*- gene expression was noted only in later stages of biofilm formation. Also, Shemesh et al. showed with planktonic *S. mutans* GS5 cells that xylitol increased the expression of *gtfB*, *gtfC,* and *gtfD* only in the late exponential growth phase in tryptone-yeast extract medium [32].

Our earlier study with an end-point biofilm model suggested that the biofilm inhibition was based on growth inhibition since the biofilms formed by the xylitol-sensitive *S. mutans* strains were inhibited by xylitol but those of xylitol-resistant strains not [19]. The results of the present study suggest that while growth inhibition may contribute to the early stages of biofilm inhibition found for both xylitol and erythritol, generally a decrease in the polysaccharide synthesis may best explain the decrease in the biofilm formation in association with these polyols. However, the lower signals of biofilm formed by strain NCTC 10449 in the presence of xylitol compared to erythritol, measured in the real-time biofilm assay, cannot be explained by the CFU-values or total polysaccharide contents, since they were similar for both polyols. The changes in impedance, or CI, measured by xCELLigence system is dependent not only on the number of attached cells or the amount of biofilm but, according to the manufacturer, also on the attachment quality of the substances on the surface of the E-plate [33]. It can thus be that also the composition, and thereby the structure and attachment strength, of the biofilm matrix is affected by the polyols, and these effects differ between xylitol and erythritol. Furthermore, the xylitol/erythritol-associated plaque reductions seen in clinical studies [8, 9, 17] may reflect a change in tightness of the biofilm attachment.

Recently, studies have suggested that antimicrobial complexes of zinc and sugar alcohols could penetrate *S. mutans* biofilms [34, 35]. In these experiments the erythritol-zinc-complex had a higher bactericidal activity compared to xylitol-zinc [34]. The same group showed also that erythritol and xylitol had a similar synergistic effect with betaine, a twitter ion, in detachment of *S. mutans* biofilms [35]. These results indicate that xylitol and erythritol may have far wider biofilm-related applications than only being biofilm formation inhibitors.

## Conclusion

Our results show that both xylitol and erythritol inhibited real-time biofilm formation by different strains of *S. mutans*. Sensitivity of the strains to polyols varied markedly, and some strains were more sensitive to xylitol and some to erythritol. Possibility to follow biofilm formation in real-time provided novel information of the effects of the polyols on the dynamics of biofilm formation. Impedance measurement also showed reduced total amount of measured biofilm for some strains. The inhibition of biofilm formation was only partly explained by a decrease in the number of viable *S. mutans* cells or the amount of polysaccharides in the biofilms, suggesting that not only was the proliferation rate of the biofilm decreased, but also the tightness of its attachment may be altered by the polyols.

## Methods

### Bacterial strains and culture conditions

Strains of *Streptococcus mutans* used: type strains NCTC 10449, NG8 and Ingbritt and the clinical isolates 113-s12, 117-s3, 124-s7, 195-s2, 199-s6, and 2366 [28]. For the biofilm assays the bacteria were grown overnight in Brain Heart Infusion Broth (BHI, Becton Dickinson, France) at 37 °C. On the day of the experiments, overnight grown bacteria were diluted 1:10 to fresh BHI and grown to mid log-phase (OD_550_ = 0.5). The log-phase bacteria were further diluted 1:50 in BHI containing 1% (w/v) sucrose (BHI-sucrose) and used for biofilm assays.

### Biofilm formation

Real-time biofilm assay was performed with an xCELLigence RTCA DP instrument (ACEA Biosciences Inc., CA, USA, [26, 36]) where biofilm forming bacteria attach to electrode sensor surface of the well in a 16 well electronic microtiter plate (E-plate). This attachment alters the measured impedance. Cell-sensor impedance is expressed as an arbitrary unit called cell index (CI). The magnitude of the change in the impedance is dependent on the number of attached cells, the size of the cells and the quantity and quality of the cell substrate e.g. biofilm matrix.

In order to study the effect of the xylitol and erythritol on biofilm formation by *S. mutans* different amounts of the two polyols were added in the growth medium and the biofilm formation was followed. First, BHI-sucrose medium (50 μl) was added to each well of 16-well E-Plate (ACEA Biosciences Inc) for background measurement, and 100 μl of cell- BHI-sucrose suspension was added. The plates were connected to the RTCA DP-system placed in CO_2_ incubator at 37 °C, and biofilm formation followed at 15-min intervals. After 1 h incubation, the run was paused and 50 μl of BHI-sucrose or BHI-sucrose supplemented with 4, 8% or 20% (w/v) xylitol (Sigma Aldrich, USA) or erythritol (Sigma Aldrich) was added and biofilm formation was again followed at 15 min intervals. Addition of medium with erythritol/xylitol induced a rapid increase in impedance signal in RTCA that slowly settled to slightly higher level compared to levels of BHI-sucrose signal. To be able to compare results obtained in different medium, the analysis program of the RTCA DP instrument (RTCA software 2.0.0.1301) was used to set the CI value at 2.5 h as one in all wells, and the results are expressed as delta CI, i.e. CI change compared to the CI at 2.5 h time point. Based on the preliminary test with different amounts of xylitol/erythritol, 20% (final concentration 5%) was selected for further assays. Biofilm tests were run in four to eight replicates and experiments repeated at least twice.

### Biofilm characterization

Biofilm formation was followed in real-time in three parallel E-plates in the RTCA equipment. At indicated time points, the run was paused and one plate per time point was removed and used either to quantify the number of viable bacteria, the amount total carbohydrate or to evaluate the expression of *gbpB*, *gtfB*, *gtfC*, *gtfD* genes in biofilm. The formation of the biofilm in the other plates was further followed.

### Colony forming units

Counts of viable *S. mutans* NCTC 10449 and 2366 cells were determined at 3, 5, 7 and 10 h biofilms. All the culture medium was carefully removed from the wells, the remaining biofilm was washed twice with phosphate buffered saline (PBS, pH 7.2) after which 100 μl of PBS was added in the well and the biofilm was detached using micro brush (Quick-Stick, Dentsol AB, Sweden) as previously described [19]. Both the micro brush and PBS were transferred to a tube containing 0.9 ml tryptic soy broth (Becton Dickinson) with 10% glycerol. Transport tubes were stored at − 20 °C before analysis. For culturing the tubes were thawed, mixed and mildly sonicated. Serial dilutions were made in saline, plated to mitis salivarius agar plates (Becton Dickinson) which were incubated 2–3 days in 7% CO_2_ atmosphere at 37 °C. The results were expressed as log CFU (colony forming units)/ml. Samples were run in triplicates.

### Polysaccharide analysis

Total polysaccharide amount in the biofilms, formed by *S. mutans* NCTC 10449 or the clinical isolate 2366, were measured at 5, 7 and 10 h by Anthrone method [37, 38] with some modifications. Briefly, the biofilm was washed twice with PBS and suspended to 100 μl of PBS with the micro bruches (Quick-Stick), as described above. Equal volume of 0.8 M NaOH was added in the biofilm suspension, mixed carefully and centrifuged 10,000 *g*, 10 min. The supernatant was diluted 1:5 with distilled water and used for analysis. An aliquot of 100 μl of the sample was mixed with 300 μl anthrone-sulfuric acid reagent (10 mg of Anthrone per 10 ml of sulfuric acid) on ice. After 10-min incubation on ice samples were boiled for 20 min and absorbance at 620 nm was measured after samples were cooled to room temperature (20–22 °C). Obtained absorbance was compared to standard curve derived from samples with known amount of dextrose. Polysaccharides were measured from 5 to 6 replicates and the amount of the carbohydrates in the biofilm suspension (μg/ml) were calculated.

### Gene expression

The gene expression in the xylitol and control biofilms of clinical isolate 2366 was evaluated at seven and 10 h. Medium was removed from the wells and the biofilms were briefly rinsed twice with warm PBS, and micro brushes (Quick-Stick) were used to suspend the biofilm in 200 μl of RNAprotect Bacteria Reagent (Qiagen, Germany) according to manufacturer’s protocol. Samples were stored at − 70 °C before processing as described previously [39].

In short, total RNA of the samples was extracted by beating with 0.1 mm glass beads (BioSpec products, OK, USA) followed by RNA purification using the Genejet RNA kit (Thermo Scientific, MA, USA). The contamination of genomic DNA was removed using the TURBO DNA-free Kit (Life Technologies, Carlsbad, USA). cDNA was synthesized with a RevertAid First Strand cDNA Synthesis Kit (Thermo Scientific, MA, USA). The expression of gbpB, gtfB, gtfC, and gtfD was examined with gene-specific PCR primers using SYBR-green based quantitative PCR (qPCR) in a Lightcycler 480II (Roche Diagnostics Nederland B.V. The Netherlands). The sequences and annealing temperature of the primers are listed in Table 1. The relative expression of each target genes was normalized by the expression of two housekeeping genes, recA and gyrA, and calculated as 2-(∆Ct), where ∆Ct = Cttarget gene-Ctgeomean of housekeeping genes). Fold changes in gene expression were further determined by 2-(∆∆Ct), where ∆∆Ct = ∆Ct of sample with xylitol-average ∆Ct of samples without xylitol [40].

**Table 1:** Primers used in this study

Gene	Primer sequence (5′- 3′)		Annealing temperature (°C)
*gbpB*	F	TCCAGCAGGGCAATGTACTTG	59
	R	CCACCATTACCCCAGTAGTTTCC	
*gtfB*	F	GACACTCCTTACCTTCAT	57
	R	TTCAGCATTATCATCAGTTC	
*gtfC*	F	GATGCTGCAAACTTCGAACA	57
	R	TATTGACGCTGCGTTTCTTG	
*gtfD*	F	TTGACGGTGTTCGTGTTGAT	59
	R	AAAGCGATAGGCGCAGTTTA	
*gyrA*	F	ATTGTTGCTCGGGCTCTTCCAG	59
	R	ATGCGGCTTGTCAGGAGTAACC	
*recA*	F	AATCACTGGCAATCTTAACTAAT	57
	R	TGATGTCCGTGGCAATAC	

### Statistical analysis

Data were analyzed with the Statistical Package for Social Science (SPSS, Version 24). The student’s t-test was performed to determine the statistical significance of the differences between sucrose, xylitol and erythritol biofilms and differences were considered statistically significant at *p* < 0.05. The gene expression data were log2 transformed prior to analysis. Student’s t-test was used to compare the fold change of each target gene in the xylitol group to the group without given xylitol at each time point. The fold change of 4 genes was compared. Therefore, the significance level (α) for Student’s t-test was adjusted to 0.0125 according to the Bonferroni correction. This, in gene expression data analysis differences were considered statistically significant at *p* < 0.0125.

## Data Availability

The datasets used and/or analysed during the current study are available from the corresponding author on reasonable request.

## Abbreviations

BHI: Brain heart infusion broth
CI: Cell index
EPS: Extracellular polysaccharide
MS: Mutans streptococci
PBS: Phosphate buffered saline
RTCA: Real time cell analyzer
